# Thiamine and Thiamine Pyrophosphate as Non-Competitive Inhibitors of Acetylcholinesterase—Experimental and Theoretical Investigations

**DOI:** 10.3390/molecules30020412

**Published:** 2025-01-19

**Authors:** Łukasz Szeleszczuk, Dariusz Maciej Pisklak, Błażej Grodner

**Affiliations:** 1 Department of Organic and Physical Chemistry, Medical University of Warsaw, 1 Banacha Str., 02-097 Warsaw, Poland; lukasz.szeleszczuk@wum.edu.pl (Ł.S.); dpisklak@wum.edu.pl (D.M.P.); 2 Chair and Department of Biochemistry and Pharmacogenomics, Medical University of Warsaw, 1 Banacha Str., 02-097 Warsaw, Poland

**Keywords:** acetylcholinesterase, acetylcholinesterase inhibitors, non-competitive inhibition, thiamine, thiamine pyrophosphate

## Abstract

Vitamin B_1_ (thiamine) plays an important role in human metabolism. It is essential for the proper growth and development of the body and has a positive effect on the functioning of the digestive, cardiovascular, and nervous systems. Additionally, it stimulates the brain and improves the psycho-emotional state. In vivo, vitamin B_1_ occurs in free form as thiamine or as its ester with phosphate residue(s), i.e., as mono-, di-, or triphosphate. It has been proven that supportive therapy with vitamin B_1_ can not only provide neuroprotection but also has a positive effect on advanced neurodegenerative diseases, such as Parkinson’s disease, Alzheimer’s disease, Wernicke–Korsakoff syndrome, or Huntington’s disease. This paper presents studies on the effect of free thiamine (T) and thiamine pyrophosphate (TPP) on the activity of acetylcholinesterase (AChE), which is an enzyme considered to play an important role in the therapies for neurodegenerative diseases, especially Alzheimer’s disease. The mechanisms of action of these compounds as potential inhibitors of AChE were evaluated using both experimental (enzymatic activity) as well as computational (molecular docking, molecular dynamics simulations, and MM-GBSA calculations) methods. The results of the current study indicate a non-competitive type of enzyme inhibition, in contrast to the previously published works suggesting a competitive one.

## 1. Introduction

Thiamine, formerly known as aneurine, is one of the earliest identified vitamins [1]. For over 70 years, it has enjoyed unwavering interest from biologists, biochemists, and physicians due to its participation in the main biochemical and physiological processes occurring in the human body [1,2,3,4,5,6,7].

**Figure 1:**
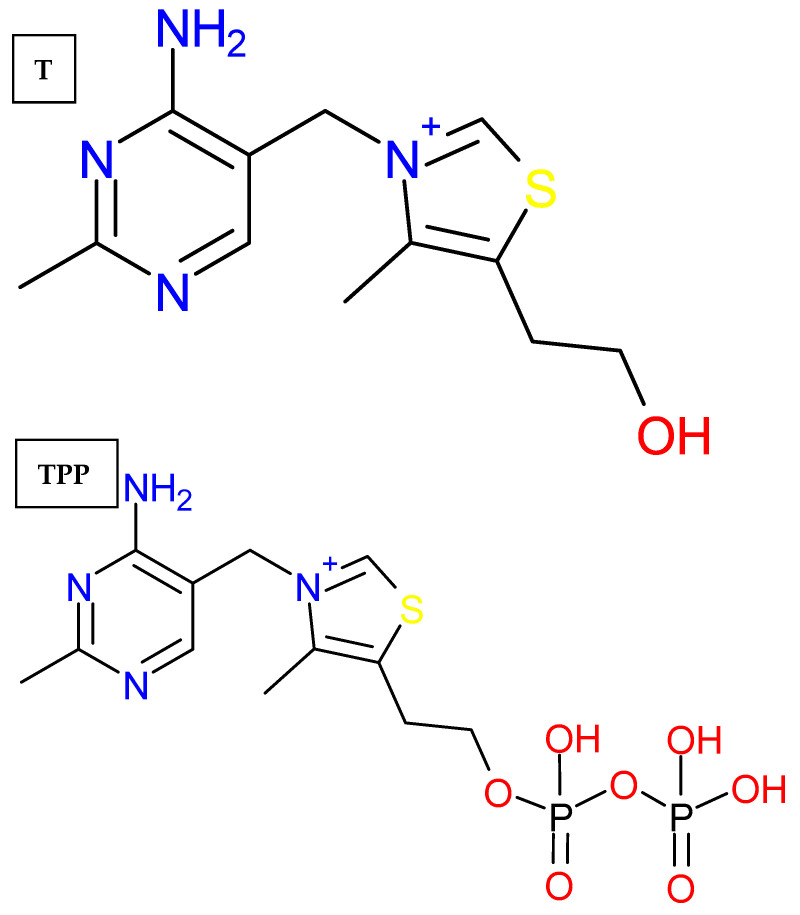
Chemical structures of thiamine (**T**) and thiamine pyrophosphate (**TPP**).

Thiamine belongs to a group of vitamins known as the B group, comprising many water-soluble compounds that differ in their chemical structures and specific functions, but also have many common features such as their important roles in cell metabolism and the synthesis of red blood cells. Thiamine occurs in large quantities in yeast, liver, groats, meat, and whole grain products. This vitamin is excreted mainly by the kidneys, and it is not stored in the body. Its characteristic feature is also that it is synthesized in small amounts by the saprophytic bacteria colonizing the human digestive tract [1,5,8]. From a chemical point of view, thiamine is composed of a substituted pyrimidine, linked by a methylene bridge with a substituted thiazole ring (Figure 1).

Free thiamine (**T**) occurs mainly in the plant world, while in animal tissues, it usually exists in the form of diphosphothiamine (pyrophosphate, **TPP**). Particularly, in the human body, thiamine occurs in three forms: monophosphate (thiamine phosphate, TMP), diphosphate (thiamine diphosphate, TDP) or pyrophosphate (thiamine pyrophosphate, **TPP**) and triphosphate (thiamine triphosphate, TTP) [2,3,4,6]. In the extracellular space, thiamine occurs as T and TMP; however, in the cytosol of the cell it occurs mostly in the form of phosphate esters. Thiamine is transported across the cell membrane in the form of TMP [9]. In commercial products, such as drugs and dietary supplements, thiamine occurs most commonly as hydrochloride, mononitrate or diphosphate [1,5,8].

Thiamine, like other group B vitamins, is an exogenous compound for humans and must be supplied with food. The daily requirement for an adult is around 2 mg. In healthy individuals, the concentration of thiamine in a serum is 0.5–1.3 µg/100 mL (14.8–38.5 nM). Only about 1% of the total body thiamine is found in the blood, 90% of which is found in erythrocytes [9]. The first symptoms of B_1_ avitaminosis usually appear after 3 weeks of not taking it in any form. It is therefore worth remembering the natural sources of thiamine in food and thus planning a properly balanced diet. The content of thiamine in food products varies greatly and ranges from 30 to 4600 µg/100 g of product [10,11].

Alzheimer’s disease is a kind of neurodegenerative disease associated with dementia. This condition is characterized by cognitive impairment, progressive memory loss, as well as a loss of the ability to think abstractly, to perform complex tasks in a logically ordered manner, and then, as the disease progresses, to perform even simple tasks [12]. The etiology of Alzheimer’s disease remains unexplained. It is certainly a heterogeneous disease, i.e., many factors contribute to its development, including age over 65. This condition is also associated with changes in the limbic system including the hippocampus and parahippocampal area, the cholinergic nucleus of the Meynert (responsible for the production of about 93% of acetylcholine in the central nervous system), and the amygdala, which participates in memory functioning [13,14]. Acetylcholine is broken down in the central nervous system by enzymes with hydrolytic properties, so-called cholinesterases. Hence, an important role in the therapy for this neurodegenerative disease is played by the drugs from the group of acetylcholinesterase (AChE) inhibitors, which inhibit the development of degenerative changes for a certain period of time, although they do not eliminate its causes.

Currently, the treatment of Alzheimer’s disease focuses on reducing the symptoms of the disease, i.e., improving cognitive and behavioral functions by normalizing cholinergic neurotransmission in the brain. Of a few theoretically possible methods of increasing transmission in the cholinergic system, such as the direct administration of acetylcholine, an increased supply of precursors, e.g., lecithin, an improved release of acetylcholine from presynaptic vesicles, the inhibition of decomposition, and direct action on receptors, only AChE inhibitors have been proven effective with relatively minor side effects [15]. A positive effect on cognitive processes, mood, and behavior, as well as a delay in the occurrence of neuropsychiatric changes have been noted during their use. The effectiveness of AChE inhibitors is explained by the stimulation of M_1_ muscarinic receptors by acetylcholine, which, by activating protein kinase C, increases the activity of α-secretase and thus prevents the formation of senile plaques [16].

As stated above, thiamine deficiency can lead to avitaminosis, of which there are two clinical types: wet and dry. The wet type is accompanied by extensive edema, causing cardiovascular disorders, circulatory failures and heart attacks. The dry type is associated with abnormalities in the functioning of the nervous system and the development of polyneuropathy and Wernicke’s encephalopathy. Thiamine deficiency is also associated with age-related disorders such as Parkinson’s disease, Alzheimer’s disease, kidney disease, cancer, mental disorders, and other diseases of the cardiovascular and nervous systems [17,18]. Supplementation with high doses of thiamine reduces the severity of Alzheimer’s disease [19] and Parkinson’s disease [20]. Many studies have also shown a direct correlation between thiamine deficiency and the symptoms of depression, which resolved after six weeks of thiamine supplementation compared to placebo [21,22,23].

The efficacy of thiamine in the treatment of Alzheimer’s disease shows promising results. Studies of oral thiamine administration have shown improvement in cognitive functions in patients with Alzheimer’s disease, but the efficacy of the therapy was dose-dependent [24,25]. It has also been shown that the concentrations of thiamine metabolites in the blood samples of patients with Alzheimer’s disease were correlated with glucose metabolism in the brain [26].

There are also some studies on the effect of thiamine on AChE activity, showing a competitive type of its inhibition [27,28]. However, in most of them, the unambiguous statement of the type of inhibition raises some controversy due to the course and location in the Lineweaver–Burk coordinate system, which should unambiguously define the type of enzymatic inhibition.

**Scheme 1:**
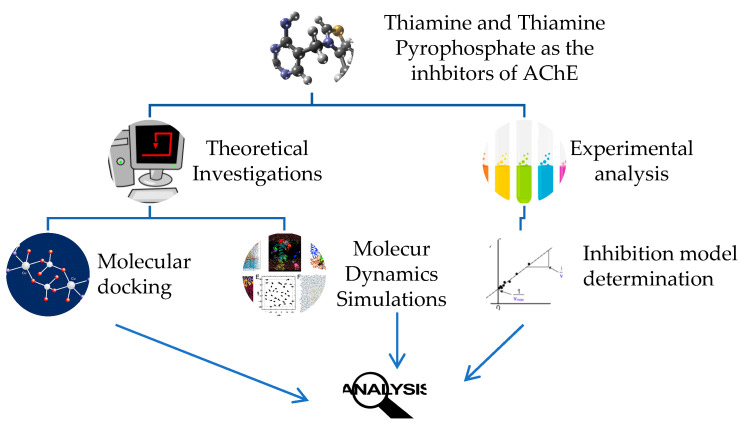
Schematic representation of the study.

Therefore, to clarify the mechanism of action of vitamin B_1_ as the acetylcholinesterase inhibitor, in this paper, we present the results of our studies using high concentrations of free thiamine (**T**) and thiamine pyrophosphate (**TPP**) on acetylcholinesterase activity. In the experimental part, we used the combined Ellman’s method to evaluate the enzymatic reaction kinetics in detail, determining the parameters such as the Michaelis–Menten constant (K_m_) and the limiting rate of the reaction (V_max_). From the variety of in silico methods, we chose molecular docking to obtain the energetically favorable complexes formed between AChE and the studied ligands, followed by the long molecular dynamic simulations and MM/GBSA calculations to assess complex stability and thermodynamics. By combining the results of the experimental and molecular modelling studies (Scheme 1), we were able to clearly determine the type of inhibition. which in both cases (**T**, **TPP**) turned out to be different from the generally postulated one.

## 2. Results and Discussion

In this study, the inhibition of enzymatic activity was analyzed in order to determine AChE inhibition by thiamine (**T**) and thiamine pyrophosphate (**TPP**).

### 2.1. Experimental Results Analysis

**Figure 2:**
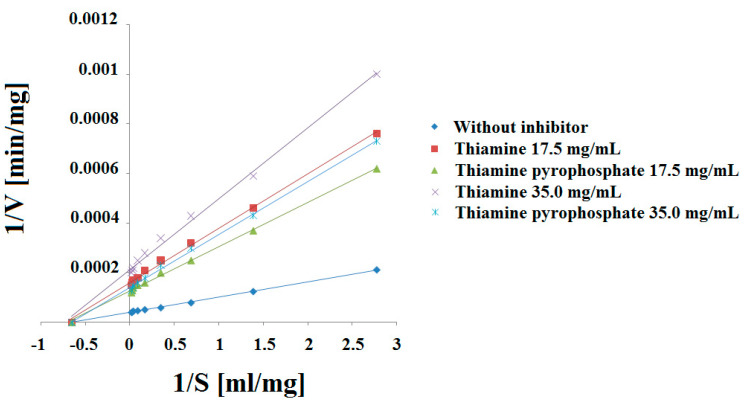
Lineweaver–Burk plots for systems with inhibitors (**T**) and (**TPP**) at concentrations of 17.5 mg/mL and 35.0 mg/mL.

When we used the combined Ellman’s method to determine the main enzyme reaction product, 4,4-dithio-bis-nitrobenzoic acid (NBA), we found the following: acetylthiocholine (ACh), 5-thio-2-nitrobenzoic acid (NTB), 5,5′-dithio-bis-2-nitrobenzoic acid (DTNB), acetylcholinesterase (AChE), and the inhibitors (**T**) and (**TPP**) of acetylcholinesterase. Nine concentrations of the reaction substrate (ACh) (0.36, 0.72, 1.44, 2.88, 5.75, 11.50, 23.00, 34.50, and 46.00 mg/mL) were employed to investigate the basic enzymatic activity when the enzyme (AChE) was present. To evaluate the impact of compounds, (**T**) and **(TPP**) inhibition, an enzymatic kinetics study was carried out in a system containing gradually increasing amounts of the substrate at two distinct concentrations (17.5 mg/mL and 35.0 mg/mL) of thiamine and thiamine pyrophosphate as the AChE inhibitors, following the earlier proposed methodology [28]. The correlation coefficient for ACh was found to be 0.9991 in the absence of inhibitors, and the slope of the curve was 0.00006. At concentrations of 17.5 mg/mL and 35.0 mg/mL for both (**T**) and (**TPP**), the correlation coefficients were within the limits, with correlation coefficients from 0.9985 to 0.9991, and corresponding curve slopes from 0.00006 to 0.00028 (Table 1, Figure 2).

The study’s initial phase involved performing kinetic tests using (**T**) and (**TPP**) to validate the AChE inhibition mechanism, postulated in the literature. The Lineweaver–Burk plots (Figure 2) illustrating the reciprocal relationship between the reaction substrate concentration (acetylthiocholine) and the reciprocal of the reaction rate were created based on the inhibition data collected in the steady-state state.

For the inhibitors (**T**) and (**TPP**) at the concentrations of 17.5 mg/mL and 35.0 mg/mL, the plots of the straight lines with different slope angles (Km/Vmax) intersected at one point on the x axis, which corresponded to the reciprocal value (Km) of the Michaelis–Menten constant. Such a mechanism is suggested by a reversible non-competitive inhibition of AChE. The calculated Km and Vmax values for these systems similarly imply a non-competitive kind of inhibition (Table 1).

The reciprocal value of the Michaelis–Menten constant (1/Km) was represented by the intersection of the plots of the straight lines with various slope angles (Km/Vmax) for the two inhibitor concentrations (**T**) and (**TPP**). A reversible non-competitive AChE inhibition suggests such a mechanism. A non-competitive form of inhibition is also suggested by the computed Km and Vmax values for the systems with varying concentrations of substances (**T**) and (**TPP**) (Table 1).

The investigation of each inhibitor’s, (**T**) and (**TPP**), binding to and inhibitory strengths in the AChE active center was the next objective of the experiment. For this reason, the values of the Michaelis–Menten constants (Km), maximum rates (Vmax), and inhibition constants (Ki) were computed for two distinct concentrations of the inhibitors (**T**) and (**TPP**) based on the obtained values of the kinetic parameters (Table 1).

The variable (decreasing) values of Vmax (3448 ± 45.7 and 2381 ± 36.4 mg/min) for the compound (**T**) and (4348 ± 42.1 and 3571 ± 37.3 mg/min) for the compound (**TPP**) and the common, similar Km values (oscillating in the range of 1.44 ± 0.05 to 1.49 ± 0.07 mg/mL) for the two inhibitors (Table 1) suggest a non-competitive type of inhibition. The lower Vmax values for the (**T**) derivative (from 3448 to 2381 mg/min) indicate that compound (**T**) is the stronger, non-competitive inhibitor of AChE, according to the initial differences in Vmax values between the individual compounds. The derivative (**TPP**) was the weaker, non-competitive inhibitor (Vmax value from 4348 to 3571 mg/min).

The angles of the Lineweaver–Burk straight lines were the next values considered to calculate the inhibition force; their values rose as the concentrations of the inhibitors (**T**) and (**TPP**) increased. For both 17.5 mg/mL and 35.0 mg/mL concentrations, the derivative (T) had the larger slope angles (11.46 × 10^−3^° ± 4.72 × 10^−5^ and 16.04 × 10^−3^° ± 3.86 × 10^−5^), whereas the derivative (**TPP**) had smaller slope angles (9.17 × 10^−3^° ± 4.83 × 10^−5^ and 10.90 × 10^−3^° ± 4.78 × 10^−5^). The derivative (**T**) was the stronger, non-competitive inhibitor of AChE, while the derivative (**TPP**) was the weaker, according to the comparisons of the Lineweaver–Burk angles for two inhibitor concentrations of the (**T**) and (**TPP**) compounds (Table 1, Figure 2).

Next, using the inhibitors (**T**) and (**TPP**) at successively higher doses, the inhibition constants (Ki) were computed (Table 1).

A stronger binding of the inhibitor (**T**) to the AChE active site was observed in the Ki values (0.2384 ± 0.006 and 0.1568 ± 0.004 mg) obtained at two concentrations (17.5 mg/mL and 35.0 mg/mL) when compared with the Ki values (4348 ± 42.1 and 3571 ± 37.3 mg) for the inhibitor (**TPP**) for the same concentrations (17.5 mg/mL and 35.0 mg/mL) (Table 1). Thus, the compound (**T**) is the more potent AChE inhibitor, whereas the compound (**TPP**) was the less potent AChE inhibitor.

By comparing the values of the Michaelis–Menten constants (Km) obtained in this study for thiamine (Km = 1.49 mg/mL) (**T**) and thiamine pyrophosphate (Km = 1.47 mg/mL) (**TPP**), with the Km values for Donepezil (Km = 1.07 × 10^−7^ mg/mL) [29] and Physostigmine (0.03 mg/mL) [30], it can be stated that thiamine has a higher affinity for acetylcholinesterase in comparison with thiamine pyrophosphate. However, after comparing the Km constants for (**T**) and (**TPP**) with the Km constants for Donepezil and Physostigmine, it is clear that thiamine has a 139,252 times weaker affinity for AChE than Donepezil and a 49.6 times weaker affinity than Physostigmine. On the other hand, **TPP** has a 137,383 times weaker affinity for AChE than Donepezil and a 49 times weaker affinity than Physostigmine.

### 2.2. Conclusions of Experimental Results

In our studies on the enzymatic kinetics of AChE under the influence of thiamine and thiamine pyrophosphate, we determined all the parameters enabling a precise determination of the type of inhibition based on the recorded kinetic changes. In the available literature, there are studies indicating or suggesting a competitive [27,28] or mixed type of inhibition [31] caused by thiamine and some other derivatives. However, the results of the aforementioned studies raise small controversies resulting from the difficulties of an unambiguous interpretation of the results. The detailed studies conducted by us and the results obtained on their basis clearly indicate a different type of inhibition. According to our studies, both thiamine and thiamine pyrophosphate show a decidedly non-competitive type of inhibition both at low and high concentration of the inhibitors used. Starting from the calculated values of the inclination angles of the Lineweaver–Burk line, the values of which increased with the increase in the concentrations of the individual inhibitors, a clear inhibition of AChE was demonstrated with respect to both inhibitors. The confirmation of the non-competitive type of inhibition is the obtained Km and Vmax values given in Table 1. Here, a constant Km value is clearly visible with the significantly changing Vmax values, which clearly indicates a non-competitive type of AChE inhibition by thiamine and thiamine pyrophosphate. In this study, we also calculated the Ki values, thus demonstrating the stronger affinity of thiamine for AChE in comparison with thiamine pyrophosphate. We also showed that both thiamine and thiamine pyrophosphate are almost 140,000 times weaker AChE inhibitors than Donepezil, but only 49 times weaker than Physostigmine. Additional confirmation of our results is shown in the molecular docking and molecular dynamics simulation analyses, conducted by us and presented below.

### 2.3. Molecular Modeling Analysis

#### 2.3.1. Molecular Docking Studies

**Figure 3:**
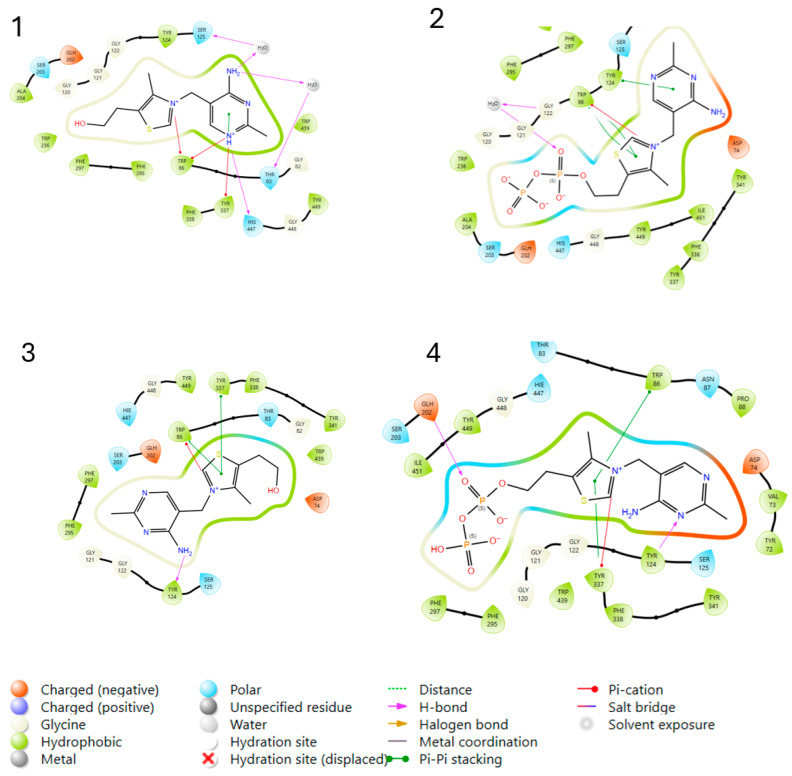
Binding modes of **T** and **TPP** with AChE obtained from molecular docking. Description of the models (1–4) is presented in Table 2.

Molecular modeling techniques were employed to obtain insight into the molecular mechanisms of interaction of **T** and **TPP** regarding the inhibition of the analyzed molecular target, AChE enzymes (EC 3.1.1.7). As the first stage of this computational analysis, molecular docking was carried out to assess the possibility of binding with the enzyme and to predict the potential bioactive conformations. Two X-ray crystal structures of human AChE (4EY6, 7XN1), deposited in the PDB database, were selected as the targets. These structures were characterized by the presence of inhibitors classified as competitive (galantamine) [32] and non-competitive (tacrine) [33] ones. Those two distinct structures were selected as our study was aimed at the possibility of an in silico assessment of the type of inhibitory activity of the analyzed compounds (**T**, **TPP**). The docking of both analyzed compounds to those two crystal structures showed that for the protein structure complexes with a non-competitive inhibitor (tacrine), significantly higher values of the scoring function were obtained. To be precise, in the case of both studied ligands, the scores were close to 11, with a slightly higher score (11.055) obtained for thiamine pyrophosphate (**TPP**). In the case of docking to the protein structure crystallized with galantamine, a competitive inhibitor, the values of the scoring function were significantly lower, which suggests that such a binding model is less favorable from the protein–ligand interaction point of view. The docking results are presented in Table 2. The binding modes presenting the ligand–protein interactions for all four complexes are depicted in Figure 3.

#### 2.3.2. Molecular Dynamics (MDs) Simulations Setup

In further studies, the interaction models (1–4) resulting from the molecular docking were used as input structures for the molecular dynamics (MDs) simulations. This approach aimed to assess the stability of the formed complexes as well as to refine the obtained results and predict the effect of the conformational matching of both interacting molecules. The MD simulations enable one to consider the dynamic effects under physiological conditions, allowing for an analysis of the stability of the complex as well as the conformational changes in both the protein and ligand due to their interaction. Simulations of 100 ns were conducted for all four models in water at 300 K temperature and 1 atm of pressure, following neutralization in an environment of 150 mM NaCl, which was supposed to reflect physiological conditions. For each of the proposed interaction models, MD trajectories were determined, with simulations **1** and **2** corresponding to the interaction with the competitively inhibited model of the enzyme and simulations **3** and **4** with the non-competitively inhibited model.

#### 2.3.3. Molecular Dynamics (MDs) Simulations Analysis

**Figure 4:**
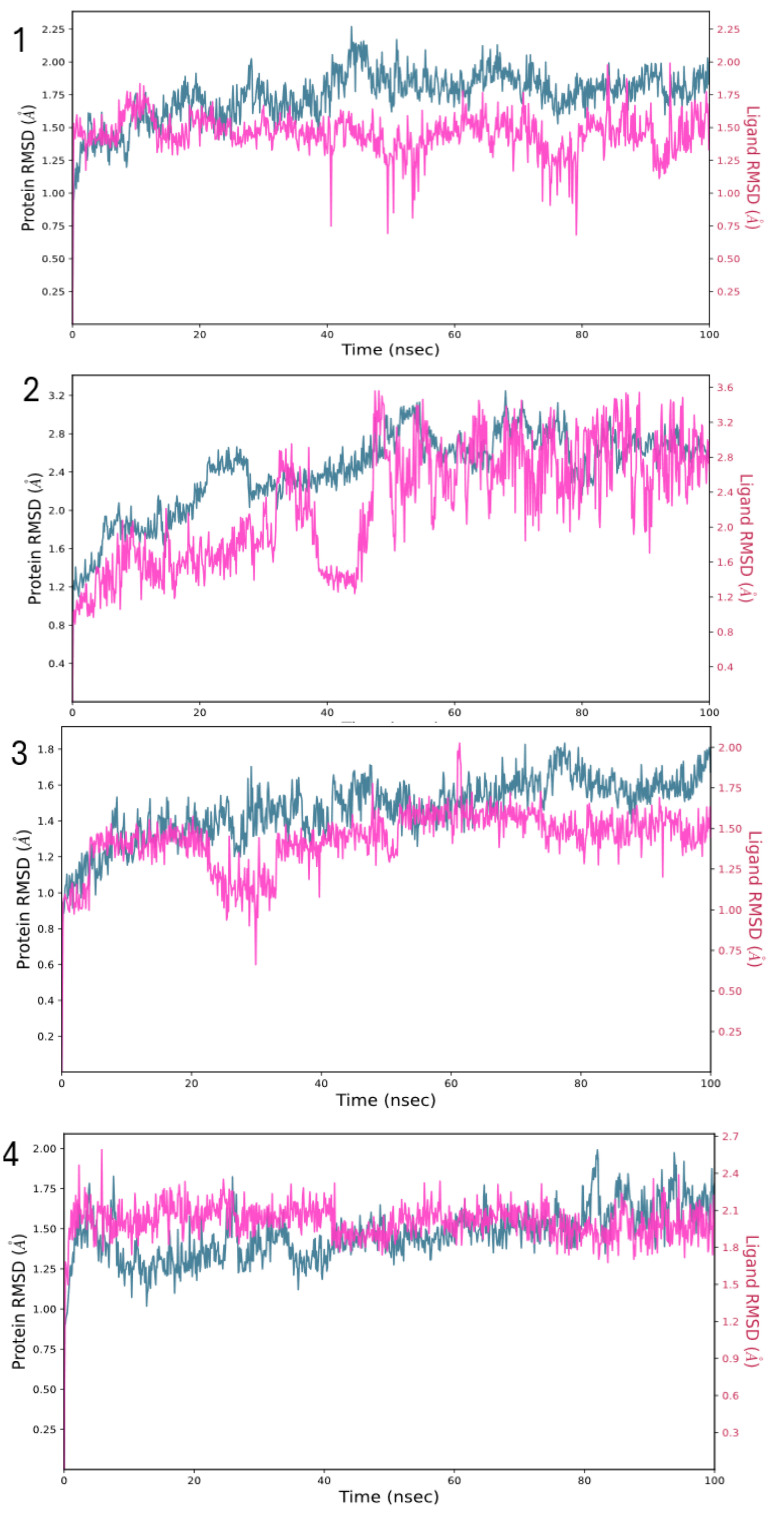
RMSD values obtained from the MD simulations. Cyan color represents the protein RMSD while pink color represents the ligand RMSD. Description of the models (1–4) is presented in Table 2.

**Figure 5:**
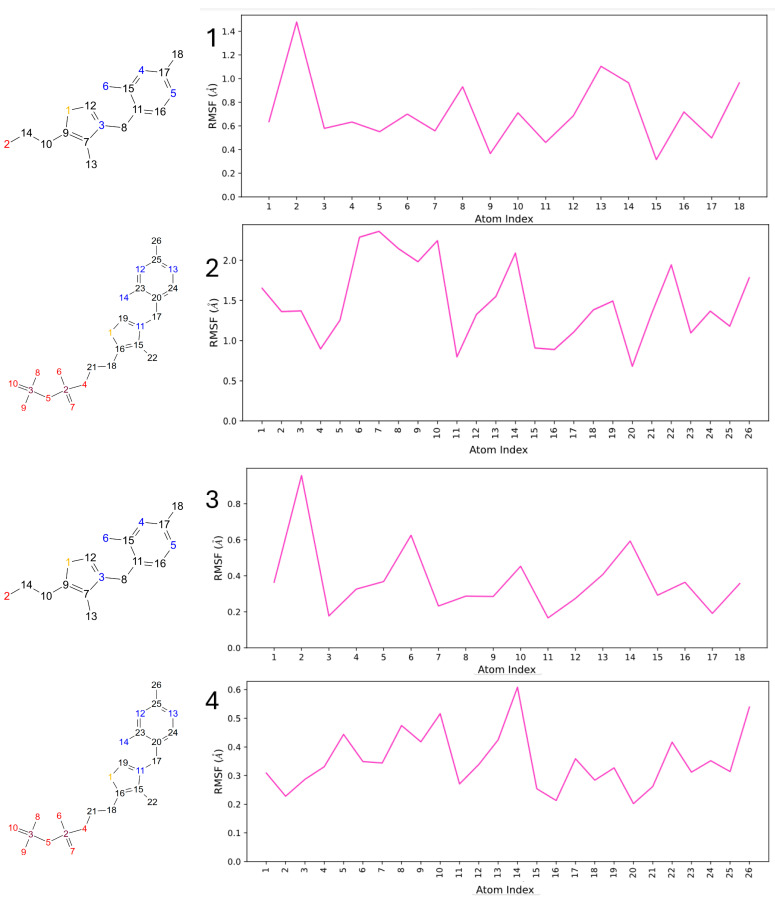
RMSF values obtained from the MD simulations. Black numbers represent carbon atoms, blue numbers represent nitrogen atoms, red numbers represent oxygen atoms, yellow numbers represent sulfur atoms, brown numbers represent phosphorus atoms. Description of the models (1–4) is presented in Table 2.

**Figure 6:**
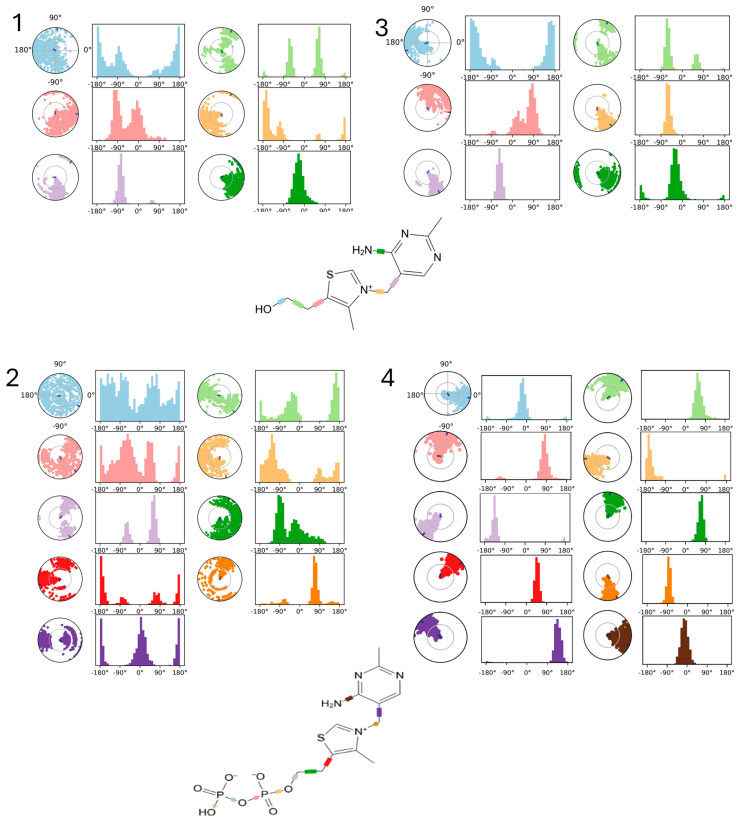
Distribution of the ligand’s torsion values, obtained from the MD simulations. Description of the models (1–4) is presented in Table 2. The colors used in the graphs represent the torsion angles indicated in the corresponding molecular structures, below the graphs.

In the MD simulations, an RMSD parameter is commonly used to assess the conformational stability of a protein ligand complex over time, by comparing the positions of the atoms to a reference structure. For simulations **1**, **3**, and **4**, stable RMSD dependencies were obtained, the exception being simulation **2** (Figure 4). Simulation **2**, which had not only significantly higher RMSD values for both the protein and the ligand in comparison to the three other simulations, was also characterized by high fluctuations of this value. Also, abrupt changes in RMSD values were observed during the simulations, which indicate a significant conformational reorganization and the considerable conformational dynamics of the ligand (**T**) in the protein pocket. This was also confirmed by the RMSF analysis (Figure 5), which served to quantify the flexibility and movement of the atoms in the selected fragments of the simulated molecular system. The RMSF parameter of the ligand was significantly larger in simulation **2**, its high values for the atoms forming the pyrophosphorous group suggest that this fragment is characterized by high conformational dynamics in this model of interactions. This was also confirmed by the analysis of the ligand torsion angle fluctuations (Figure 6), showing a significant conformational freedom for the phosphate group and the ethylene chain in simulation **2**, compared to simulation **4** with the same ligand.

The molecular dynamics simulations enabled the study of the interactions between the ligands and proteins that are responsible the stability of those complexes. It has been shown that π-π interactions and hydrogen-bond interactions with the nitrogen atoms in the aromatic rings of thiamine are the main factors stabilizing the formed complexes. For simulation **3,** conducted for the **T** bonded with AchE in the non-competitive mode, their fundamental role in stabilizing the complex is shown in the π-π interaction of TRP86 and the hydrogen bonds formed by amino acids such as TRP 86, SER203, and TYR337 with the heterocyclic rings of the ligand, as well as the ASN87 interacting with the hydroxyl group of the ethanolic moiety of **T**. For simulation **4**, performed for **TPP**, the complex is stabilized by the π–π interactions with TRP86, and the hydrogen-bond interactions with TYR72, ASP74, and SER125. Additionally, the presence of a polar pyrophosphorous group allows for the formation of numerous hydrogen bonds with amino acids such as GLU202, TYR 337, HIS 447, and SER 203. These bonds were often transferred by water molecules, forming water bridges.

**T** and **TPP** were found to interact with multiple residues. The exception was in simulation 2 of the interaction of **TPP** with the AChE structure, originally bonded with the competitive inhibitor of this enzyme. The stability of this complex was mainly related to the interaction of the phosphate group of **TPP** with TYR 337. This was consistent with the analysis of the dynamics of this complex, as the large conformational freedom suggested a lack of strong interactions stabilizing the bioactive conformation in the active site.

Additionally, an analysis of the trajectory revealed that the presence of a pyrophosphate group allows for the creation of an intermolecular hydrogen bond within the molecule. Although rarely seen in simulation 2, the interaction can be observed frequently in simulation 4, stabilizing the ligand’s conformation in the binding site and reducing the energy of the complex.

**Figure 7:**
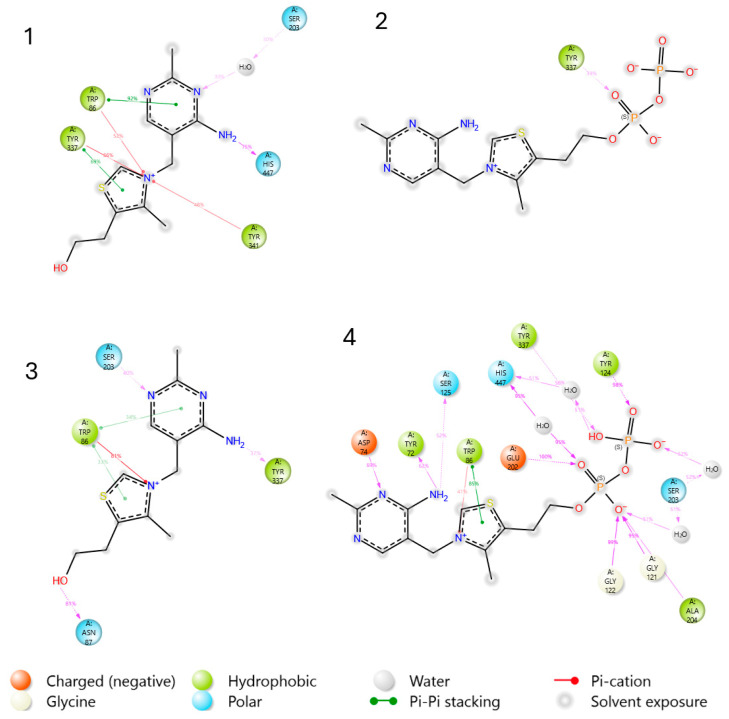
Binding modes of T and TPP with AChE obtained from molecular dynamics. Description of the models (1–4) is presented in Table 2.

**Figure 8:**
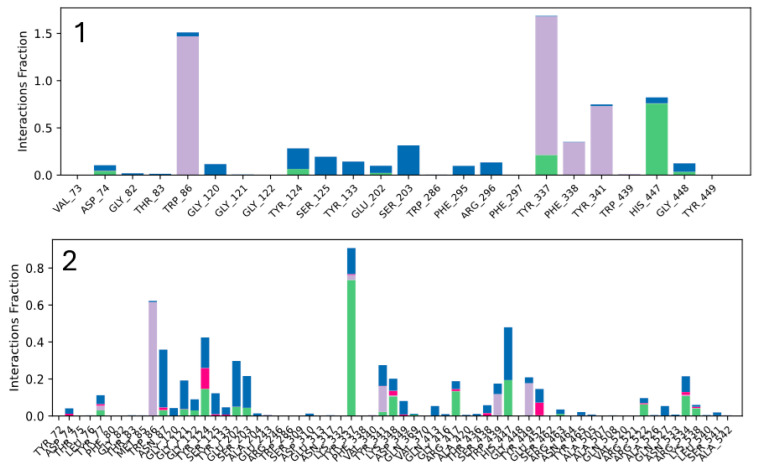

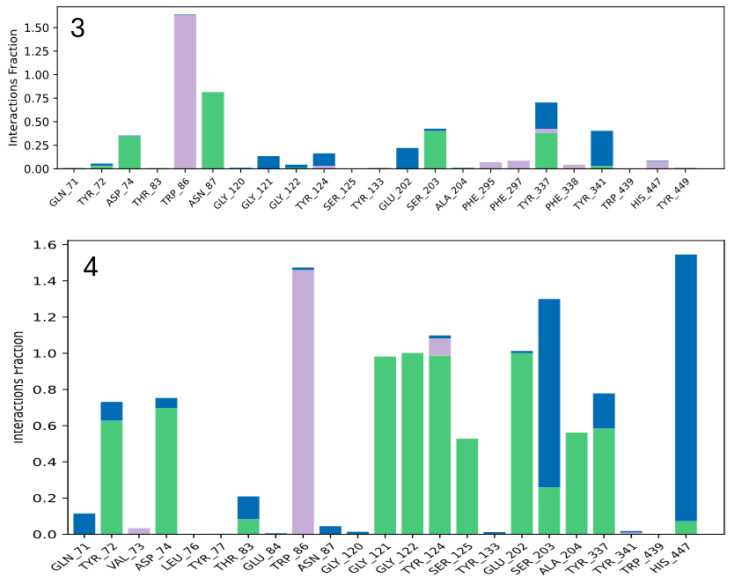
Interactions of ligands, T, and TPP, with AChE obtained from molecular docking. Description of the models (1–4) is presented in Table 2. The stacked bar charts are normalized over the course of the trajectory; for example, a value of 0.7 suggests that 70% of the simulation time for the specific interaction is maintained. Values over 1.0 are possible as some protein residue may make multiple contacts of same subtype with the ligand. Green color represents H-bonds, violet color represents hydrophobic interactions, pink color represents ionic interactions, blue color represents H-bonds through water bridges.

The diagrams showing the ligand–enzyme interactions for the individual simulations are presented in Figure 7 and Figure 8.

#### 2.3.4. MM-GBSA Calculations

In the last stage of the computational part of this study, in order to increase the reliability of the obtained results, MM-GBSA calculations of the free energy of the interactions were conducted. MM-GBSA is a widely used computational method for estimating the binding free energy of molecular complexes by combining molecular mechanic force fields with continuum solvation models. The approach involves calculating the gas-phase energy, solvation energy, and surface area contributions to obtain the total binding free energy, offering insights into the molecular interactions that drive ligand–receptor binding. The MM-GBSA values for 500 frames from the last 50 ns of the simulations were calculated and then averaged. The results are presented in Table 3.

The free energy values obtained were similar to the docking scores (Table 2), and notably higher for the AChE crystallized with the non-competitive antagonist. The significant reduction in estimated free energy values is readily observable with the pyrophosphate group present in the ligand.

#### 2.3.5. Molecular Modeling Conclusions

The in silico results show that the mechanism of inhibition for both **T** and **TPP** corresponds to the non-competitive type of inhibition. At the same time, the obtained results suggest that **T** is characterized by a higher affinity for the active site of AChE than **TPP**, which is also consistent with the experimental results from our study. The analysis of the ligand–enzyme interactions suggests that the key contributions to the stabilization of the formed complexes are the π–π interactions of the aromatic rings of TRP86 and thiamine, as well as the hydrogen bonds formed between the amino group and nitrogen atoms in the pyridazine ring of the ligands and various residues.

## 3. Materials and Methods

### 3.1. Experimental Studies

#### 3.1.1. Materials

The human acetylcholinesterase (EC. 3.1.1.7, AChE), acetylthiocholine iodide (ACth), 5,5′-Dithiobis(2-nitrobenzoic) acid (DTNB), thiamine (**T**), and thiamine pyrophosphate (**TPP**) were obtained from Sigma-Aldrich (Poznań, Poland). The studies were carried out with the use of the Shimadzu UV-Vis-NIR Spectrophotometer UV-3600 Plus (Kyoto, Japan).

#### 3.1.2. Working Solutions

After dissolving the AChE in a phosphate buffer (pH 7.5), it was separated into multiple pieces and kept at −20 °C for storage. Every day, one aliquot was thawed, and AChE activity was monitored. Just before usage, the necessary concentrations of the DNTB and ACth working solutions were made by diluting them with the phosphate buffer. Additionally, primary stock standard solutions for ACth were made independently using deionized water. A concentration of 46.00 mM was achieved by dissolving 332.5 mg of ACth in 25 mL of the phosphate buffer (100 mM, pH 7.5). After diluting the produced ACth solution with 100 mM of the phosphate buffer, mixed working standard solutions with concentrations of 34.50, 23.00, 11.50, 5.75, 2.88, 1.44, 0.72, and 0.36 mg/mL were obtained.

#### 3.1.3. Method

**Figure 9:**
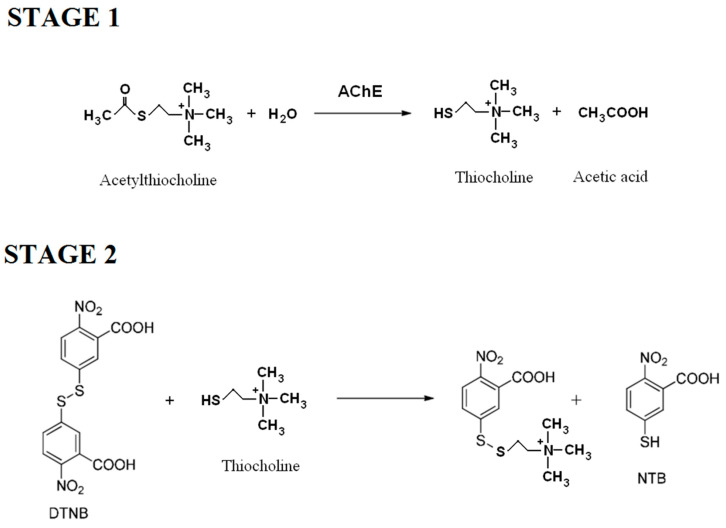
Reaction scheme for the determination of acetylcholinesterase activity [35].

The spectrophotometric approach of Ellman et al. [34], which uses 5,5′-dithiobis-(2-nitrobenzoic acid) (DTNB) as the thiol reagent and thiocholine esters as substrates, is a commonly used technique for measuring acetylcholinesterase activity. It is predicated on the interaction of DTNB or Ellman’s reagent with thiocholine, which is generated by the enzymatic hydrolysis of the synthetic substrate ATCh. By tracking absorbance at 412 nm, the yellow result of this reaction, 5-thio-2-nitrobenzoic acid (TNB), is formed. Ellman’s approach [34] was used to assess the activity of acetylcholinesterase, although it was somewhat modified and previously reported in the prior study [35]. Briefly the approach was as follows: the reaction mixture (2 mL final volume) was composed of 1.6 mL of 100 mM of the phosphate buffer pH (7.5), 0.1 mL of 4 mM of 5,5′-dithio-bis-2-nitrobenzoic acid (DTNB), and 35.0 mg and 70.0 mg of T or TPP were directly dissolved in 2 mL of the reaction mixtures. The method is based on the formation of a yellow anion, 4,40-dithio-bis-acid nitrobenzoic, measured by absorbance at 412 nm, during 3 min of incubation at 25 °C. The enzyme was pre-incubated for 2 min. The reaction was initiated by adding 0.1 mL of 200 mM of acetylthiocholine iodide. The method was based on two stages (Figure 9).

The free thiocholine (stage 1), formed from acetylthiocholine under the influence of AChE, reacts then with the DTNB releasing a certain amount of NTB (stage 2), the concentration of which depends on the AChE activity [35].

The results obtained from determining the inhibition type using the following equation are shown on the Lineweaver–Burk coordinate system in Figure 2 and Table 1:

(1) $$\frac{1}{V}=\frac{K_{m}}{V_{max}\left[S\right]}+\frac{1}{V_{max}}$$

### 3.2. Molecular Modelling

#### 3.2.1. Software Used

All computations were performed utilizing various modules (LigPrep, Epik, Glide, Prime, Desmond) of the Schrödinger Suite via the Maestro graphical interface (Schrödinger Release 2021–1).

#### 3.2.2. Ligands Preparation

The ligand structures utilized in this study, comprising thiamine (ID: 1130) and thiamine pyrophosphate (ID: 9068), were obtained from the PubChem database [36]. A thorough prediction of all potential tautomeric and ionization states, stereoisomers, and ring conformations is a crucial stage in ligand docking. To ensure a precise 3D model creation and a conformational sampling of the ligands, both ligands underwent structural refinement and optimization with the LigPrep package, employing the OPLS4 force field. Protonated states were produced at pH 7.0 ± 1.0 using the Epik module.

#### 3.2.3. Protein Preparation

Atomic coordinates from the crystal structures of AChE complexed with the competitive inhibitor galantamine (PDB 4EY6) and the non-competitive inhibitor tacrine (PDB 7XN1) were obtained from the Protein Data Bank. The system was constructed with the Protein Preparation Wizard, hydrogen atoms were incorporated, and pKa values for the protein residues were computed via the PROPKA method at pH 7.4. The protein–ligand complexes underwent controlled minimization, achieving a convergence of the heavy atoms to an RMSD of 0.3 Å, utilizing the OPLS4 force field. The optimized structure was employed to create the grid for the docking computations.

#### 3.2.4. Docking Calculations

In both cases (4EY6 and 7XN1), the centroid of the corresponding ligand situated in the active site was utilized to establish a cubic grid inner box measuring 10 × 10 × 10 Å and a cubic grid outer box measuring 20 × 20 × 20 Å. The docking calculations were performed using the Glide flexible docking method, utilizing extra precision (XP) and a Glide scoring function with the OPLS4 force field. No Epik state penalties were incorporated into the final docking scores.

#### 3.2.5. Molecular Dynamics (MDs) Simulations

MD simulations were performed with Desmond under NPT conditions at T = 300 K and p = 1 atm. The Langevin thermostat and barostat were utilized, with the relaxation times set to 1.0 ps and 2.0 ps, respectively. The best scored docking poses of the protein–ligand complexes served as the initial configurations. Each docking pose was solubilized within a truncated octahedral TIP3P water box with the dimensions of 20 Å. The system was neutralized by the addition of an appropriate quantity of Cl^−^ ions, and NaCl salt (0.15 M) was incorporated to establish a physiological environment. The systems were equilibrated using the ‘desmond_npt_relax.msj’ protocol in Desmond, employing default parameters and the OPLS4 force field. The integration time step was established at 2 fs, and the total simulation time was 100 ns, with a cutoff radius of 9 Å selected for the short-range Coulomb interactions in the u-series decomposition of the Coulomb potential.

Simulation interaction diagram reports were utilized for mapping and analyzing the geometry of the ligand-binding sites and identifying the amino acid residues involved in the protein–ligand interactions. The reports encompass (i) protein data detailing the secondary structure elements; (ii) ligand data outlining the torsion angle profiles and surface properties; (iii) RMSD and RMSF visualizations; and (iv) data on the protein–ligand interactions.

#### 3.2.6. MM-GBSA Calculations

The Molecular Mechanics Generalized Born Surface Area (MM-GBSA) of the binding free energy (dG) was computed using the Prime VSGB2.0 implicit water model and the OPLS4 force field. The thermal_mmgbsa.py script was utilized to compute the MM-GBSA binding energy for frames derived from the trajectories of molecular dynamics simulations. From each trajectory, 500 frames from the last 50 ns of the simulation time were selected. The MM-GBSA calculations were conducted on these frames following the removal of water molecules and the separation of the ligand from the receptor, as implemented in the thermal_mmgbsa.py script.

## 4. Conclusions

In this study, using both experimental and computational methods, we confirmed the role of **T** and **TPP** as potent AChE inhibitors. The results of the experimental analysis undoubtedly indicate the non-competitive mechanism of action of these two ligands. Additionally, to the best of our knowledge, the comprehensive evaluation of this phenomenon at the molecular level, including the use of advanced in silico methods such as molecular dynamics simulations and MM-GBSA calculations, has been performed for the first time. Also, the combined Ellman’s method applied in this study has not been used before for this purpose.

Surprisingly, unlike the previously published results, the findings of the current study clearly demonstrate the non-competitive mechanism of action. This suggests that it may be reasonable to revisit the other inhibitors of AChE as well, and validate their mechanism of action, especially taking into account the important role of those compounds in the therapies for neurodegenerative diseases.

## Figures and Tables

**Table 1:** The analytical data describing effects of acetylcholinesterase inhibition by (**T**) and (**TPP**) and regression equation for acetylthiocholine for 6 replicates for each sample (n = 6) at the concentration range 0.36–46.00 mg, in the presence of inhibitors (**T**) and (**TPP**) at concentrations of 17.5 mg/mL and 35.0 mg/mL.

Thiamine (T)			
Concentration (mg/mL)	Straight Line Equation	R^2^	Tilt Angle (°)
(**T**) 0.00	y = 0.00006x + 0.00004	0.9991 ± 0.0015	3.44 × 10^−3^ ± 3.10 × 10^−5^
(**T**) 17.5	y = 0.00020x + 0.00029	0.9985 ± 0.0037	11.46 × 10^−3^ ± 4.72 × 10^−5^
(**T**) 35.0	y = 0.00028x + 0.00042	0.9986 ± 0.0032	16.04 × 10^−3^ ± 3.86 × 10^−5^
**Concentration** **(mg/mL)**	**K_m_ (mg/mL)**	**V_max_ (mg/min)**	**K_i_ (mg)**
(**T**) 0.00	1.49 ± 0.03	25,000 ± 78.6	---
(**T**) 17.5	1.45 ± 0.08	3448 ± 45.7	0.2384 ± 0.006
(**T**) 35.0	1.49 ± 0.07	2381 ± 36.4	0.1568 ± 0.004
**Thiamine pyrophosphate (TPP)**			
**Concentration ** **(mg/mL)**	**Straight Line Equation**	**R^2^**	**Tilt Angle (°)**
(**TPP**) 0.00	y = 0.00006x + 0.00004	0.9991 ± 0.0015	3.44 × 10^−3^ ± 3.10 × 10^−5^
(**TPP**) 17.5	y = 0.00016x + 0.00023	0.9988 ± 0.0038	9.17 × 10^−3^ ± 4.83 × 10^−5^
(**TPP**) 35.0	y = 0.00019x + 0.00028	0.9985 ± 0.0034	10.90 × 10^−3^ ± 4.78 × 10^−5^
**Concentration** **(mg/mL)**	**K_m_ (mg/mL)**	**V_max_ (mg/min)**	**K_i_ (mg)**
(**TPP**) 0.00	1.49 ± 0.03	25,000 ± 78.6	---
(**TPP**) 17.5	1.44 ± 0.05	4348 ± 42.1	0.3137 ± 0.008
(**TPP**) 35.0	1.47 ± 0.08	3571 ± 37.3	0.2483 ± 0.007

**Table 2:** Molecular docking results for the complexes formed between **T**, **TPP,** and human AChE.

Protein PDB Code	Co-Crystalized Inhibitor	Docked Ligand	Model	GLIDE XP Score
4EY6	Competitive Galantamine	Thiamine T	1	−2.465
		Thiamine pyrophosphate TPP	2	0.823
7XN1	Non-competitive Tacrine	Thiamine T	3	−10.735
		Thiamine pyrophosphate TPP	4	−11.054

**Table 3:** MM-GBSA results for the complexes formed between T, TPP, and human AChE.

Protein PDB Code	Co-Crystalized Inhibitor	Docked Ligand	MM-GBSA [kcal/mol]
4EY6	Competitive galantamine	Thiamine T	−42.57
		Thiamine pyrophosphate TPP	−9.57
7XN1	Non-competitive tacrine	Thiamine T	−63.00
		Thiamine pyrophosphate TPP	−45.44

## Data Availability

The data can be obtained from the corresponding author (B.G.) by email.
